# Internal carotid artery occlusion related to poorly controlled rheumatoid arthritis presenting with continuous hand shaking: A case report and literature review

**DOI:** 10.1097/MD.0000000000029001

**Published:** 2022-03-04

**Authors:** Ching-Fang Chien, Chun-Yi Tsai, Meng-Ni Wu, Chiou-Lian Lai, Li-Min Liou

**Affiliations:** a Department of Neurology, Kaohsiung Medical University Hospital, Kaohsiung Medical University, Kaohsiung, Taiwan; b Department of Neurology, School of Medicine, College of Medicine, Kaohsiung Medical University, Kaohsiung, Taiwan.

**Keywords:** case report, hand shaking, internal carotid artery occlusion, rheumatoid arthritis, transient ischemic attack

## Abstract

**Rationale:**

Limb-shaking syndrome is a special manifestation of transient ischemic attack, resulting from internal carotid artery (ICA) occlusion. Extra-articular manifestations of rheumatoid arthritis (RA) are likely to occur in patients with severe or active RA. RA may accelerate atherosclerotic processes through inflammation. Here, we present a case of ICA occlusion related to poorly controlled RA that presented with continuous hand shaking.

**Patient concerns:**

A 73-year-old man with a history of poorly controlled RA developed total occlusion of the right ICA in recent 4 months. He presented with 2 days of continuous and rhythmic left-hand shaking before admission.

**Diagnosis:**

The patient was suspected to have transient ischemic attack resulting from ICA occlusion.

**Interventions:**

Antiplatelets and antiepileptic drugs were used for continuous nonepileptic focal myoclonus. A disease-modifying antirheumatic drug-based regimen for RA was developed to prevent further atherosclerosis.

**Outcomes:**

Following the initial intervention, continuous hand shaking subsided on hospital day 7. Prednisolone was titrated as an active RA control. At the 6-month follow-up visit, neither painful wrist swelling nor recurrent shaking of the hand was noted.

**Lessons:**

Continuous hand shaking (nonepileptic focal myoclonus) can be the initial presentation of ICA occlusion in patients with poorly controlled RA. Every patient with RA should be treated aggressively with anti-rheumatic agents since RA is an independent risk factor for stroke. Additionally, every patient with RA should be surveyed for ICA stenosis, especially in those with poor control.

## 1 Introduction

Limb-shaking syndrome (LSS) is a rare but important presentation of transient ischemic attacks resulting from occlusion of the internal carotid artery (ICA), which is often misdiagnosed as a focal seizure. A lack of epileptiform discharge on electroencephalography (EEG) and severe ICA occlusion on carotid duplex or angiography can be used to make an accurate diagnosis. Although rheumatoid arthritis (RA), a chronic inflammatory disease, is more common in women, extra-articular manifestations of RA are more common in men, especially in those with severe and active disease.^[1]^ Furthermore, RA is associated with the development of atherosclerotic lesions and increases the risk of ischemic stroke by 64% relative to that in the general population.^[2]^ In this case report, we present a patient with poorly controlled RA without major risk factors for stroke who developed total occlusion of the right ICA over the course of 4 months and presented with continuous rhythmic left-hand shaking (nonepileptic focal myoclonus), which clinically appeared to be epilepticus partialis continua (EPC).

## 2 Case presentation

A 73-year-old male presented to our emergency department with worsening left limbs weakness and 2 days of continuous, involuntary left-hand shaking that he could not suppress. He denied any loss of consciousness. No aura was found. Hand shaking worsened after neck extension and postural changes from sitting to standing. He had a medical history of a recent ischemic stroke in the territory of the right middle cerebral artery (MCA) 4 months earlier, and cilostazol was administered for secondary prevention. Additionally, he had been diagnosed with seropositive RA 1 year ago, which manifested as pain in multiple joints. His RA was controlled with prednisolone, sulfasalazine, and methotrexate, but bilateral wrist pain and swelling were still noted in recent months. Previous sequelae of stroke included left hemiplegia (Medical Research Council scale, muscle power: 4) and dysphagia. He had no history of diabetes, hypertension, smoking, or other traditional risk factors for cerebrovascular disease.

On admission, the patient's vital signs were as follows: temperature, 36.2°C; blood pressure, 146/71 mmHg; heart rate, 83 bpm; and oxygen saturation, 99% on room air. Physical examination revealed bilateral wrist swelling, with local heat and pain. A neurological examination revealed that the patient was fully conscious. The left limbs were found to be weaker than the right limbs on the Medical Research Council scale, with a muscle power of 2 in the left upper limb, 3 in the left lower limb, and 5 in the right limbs. The shaking pattern of the left hand was a continuous, rhythmic jerky movement throughout the left hand that occurred at 3- to 5-second intervals. It was not suppressible and was continued during sleep. Shaking was initially clinically diagnosed as an EPC.

**Figure 1:**
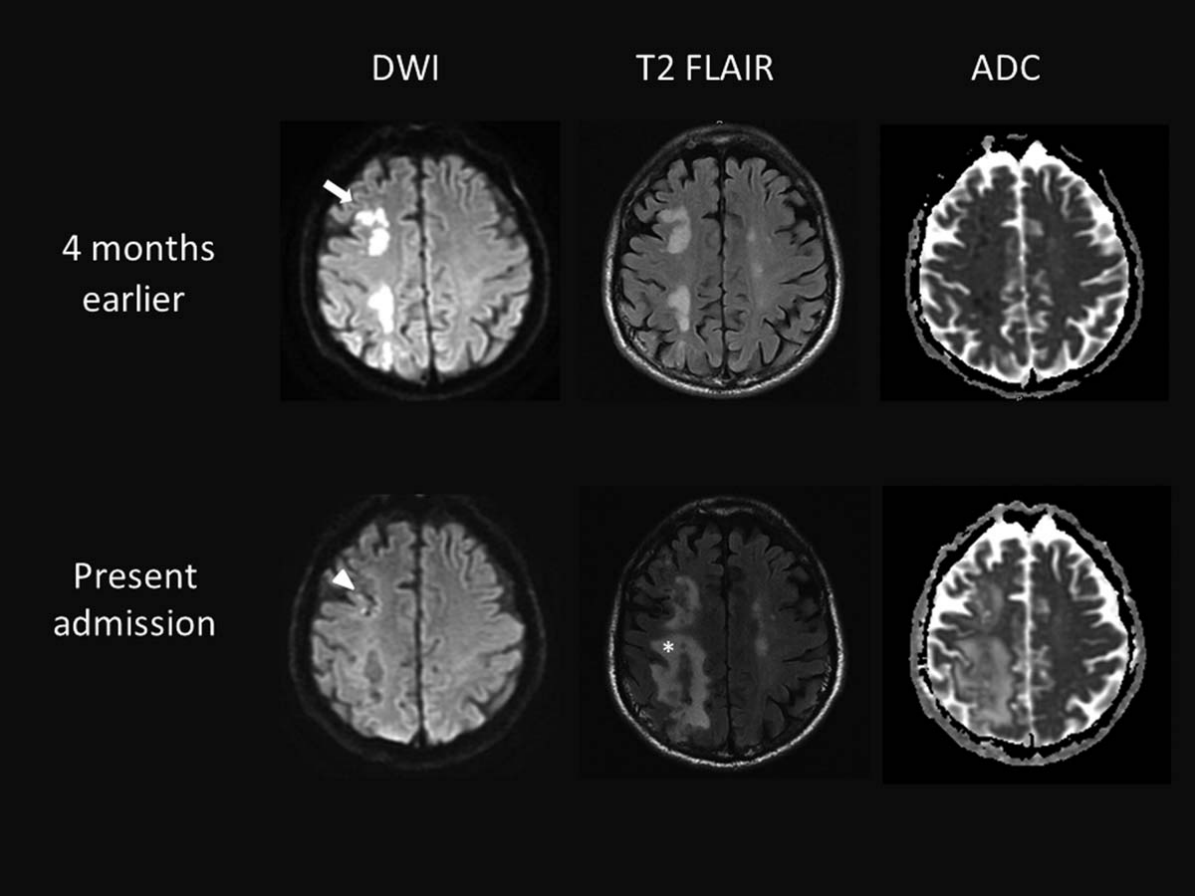
Brain magnetic resonance imaging (MRI) performed 4 months earlier revealed acute infarction in the right middle cerebral artery (MCA) territory (arrow). Brain MRI taken at the time of this admission showed multifocal increased diffusion-weighted imaging/fluid-attenuated inversion recovery signal superimposed in the right frontoparietal gyrus adjacent to the previously infarcted area (arrowhead). An increased burden of white matter hyperintensity in the right MCA territory was noted (asterisk).

**Figure 2:**
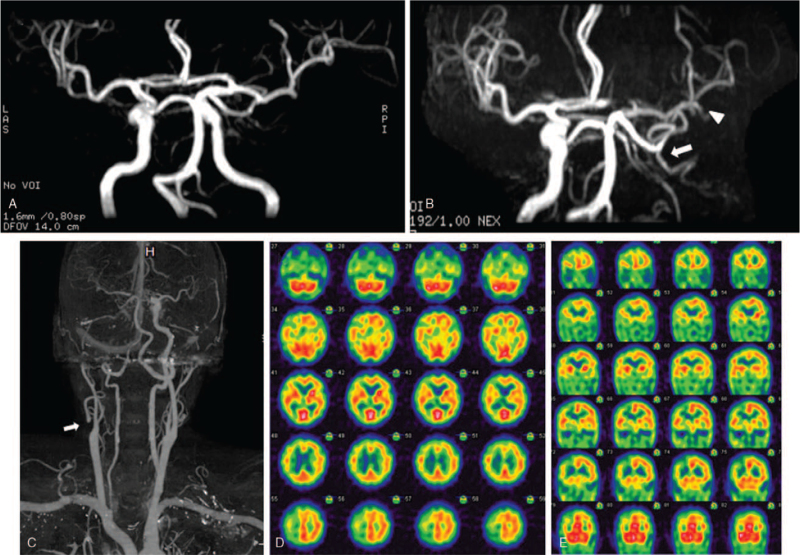
(A) Magnetic resonance angiography (MRA) taken 4 months earlier revealed patent bilateral internal carotid artery (ICA). (B) At the time of this admission, MRA revealed occlusion of the right internal carotid artery (ICA) (arrow) with resultant decreased flow in the right middle cerebral artery (MCA) (arrowhead). (C) Brain computed tomography angiography at the time of this admission revealed total occlusion of the right ICA from the carotid bifurcation to the terminus (arrow). (D, E) Tc-99m ethyl cysteinate dimer brain single-photon emission computed tomography showed hypoperfusion involving the right cerebrum, especially in the right MCA territory.

The general laboratory data were unremarkable. Autoantibody tests for rheumatoid diseases revealed a rheumatoid factor level of 252 IU/mL (normal range <15.9 IU/mL), erythrocyte sedimentation rate of 63 mm/h (normal range: 0–15 mm/h), and C-reactive protein of 24.98 mg/L (normal range <5.0 mg/L). The lipid profile showed a total cholesterol (TC) level of 162 mg/dL, high-density lipoprotein (HDL) level of 40 mg/dL, and low-density lipoprotein (LDL) level of 101 mg/dL. Electroencephalography showed focal, intermittent polymorphic theta and delta waves in the right cerebral hemisphere. Therefore, continuous left-hand shaking was more similar to non-epileptic focal myoclonus than to EPC. Brain magnetic resonance imaging obtained during hospitalization showed a multifocal increased diffusion-weighted imaging/fluid-attenuated inversion recovery signal superimposed in the right frontoparietal gyrus adjacent to the prior infarction area (Fig. 1). The bilateral MCA blood flow appeared equal four months earlier; however, magnetic resonance angiography taken at this time showed occlusion of the right ICA with resultant decreased flow in the right MCA (Fig. 2). There were fewer right MCA vessels than before, and surrounding collateral circulation developed. Additionally, infarction was still present, but was less apparent. This suggests that the blood flow in this area was relatively hypoperfused, and infarction could have recurred if hypotension was sustained. Carotid ultrasonography revealed total occlusion of the right ICA with right ophthalmologic artery reverse flow consistent with collateral circulation, whereas carotid ultrasonography and magnetic resonance angiography performed 4 months earlier revealed a patent ICA. Single-photon emission computed tomography of the brain revealed hypoperfusion in the right MCA territory.

After admission, the patient was prescribed dual antiplatelet therapy consisting of cilostazol, dipyridamole, and statins for secondary stroke prevention. For continuous non-epileptic focal myoclonus, 0.25 mg clonazepam was prescribed starting on hospital day 1 and an intravenous loading dose of lacosamide (200 mg) was administered on day 3. Starting on hospital day 4, oral lacosamide (100 mg daily) and perampanel (2 mg every night) were prescribed. The shaking subsided gradually starting on day 7 of hospitalization. Prednisolone was titrated from 5 to 20 mg/day (day 3) for active RA control. At the 6-month follow-up visit, neither painful wrist swelling nor recurrent shaking of the hand was noted.

## 3 Discussion

RA is an autoimmune rheumatic disease that affects approximately 0.5%–1% of adults worldwide.^[3]^ RA affecting extra-articular organs, including the skin, eye, heart, lung, and renal, gastrointestinal, and nervous systems, increases mortality.^[1]^ Extra-articular involvement is also more common in men and in those who are rheumatoid factor and/or HLA-DR4 positive.^[1]^ Our case was diagnosed with “definite RA” based on the 2010 American College of Rheumatology/European League Against Rheumatism (ACR/EULAR) classification criteria. He was at risk of extra-articular involvement since he was male and likely had poor control of his RA, as evidenced by persistent wrist arthritis, elevated C-reactive protein, erythrocyte sedimentation rate, and RA factor. RA is now believed to be an independent risk factor for stroke,^[4]^ and some opinions suggest that active RA could be as strong a risk factor as diabetes mellitus. However, the exact atherosclerotic effects of RA remain under investigation. Chronic inflammation that directly affects the vessel wall or dyslipidemia has been proposed.^[3]^ The atherosclerotic effects are also associated with disease activity.^[3]^ In addition, RA can result in vessel occlusion through bone erosion and arthritis. Some case reports have described infarctions secondary to atlantoaxial dislocation or subluxation.^[5,6]^

Positron emission tomography (PET) can reveal arterial inflammation in patients with active RA.^[7]^ Compared to the general population, atherosclerotic plaques of RA might be more prone to rupture.^[8]^ Most patients with RA have an abnormal lipid profile characterized by increased TC and LDL levels, reduced HDL levels, and/or an increased TC/HDL index, which is adversely affected by disease activity.^[9]^ This patient's lipid profile on admission was mildly worse than that four months earlier. Specifically, on admission, the patient had the following lipid profile: TC, 162 mg/dL; HDL, 40 mg/dL; and LDL, 101 mg/dL. Four months earlier, his lipid profile was as follows: TC, 134 mg/dL; HDL, 39.7 mg/dL; and LDL, 80 mg/dL. These lipid profile changes were comparable to those in RA patients receiving disease-modifying antirheumatic drug (DMARD) treatment. Furthermore, a lower TC level was not related to lower cardiovascular (CV) risk. A population-based cohort study, which included 651 patients with RA, found that the group with lower cholesterol (TC <155 mg/dL) showed a 3.3-fold increase in CV risk.^[10]^ These results may explain why accelerated ICA occlusion correlates with a poorly controlled RA.

Patients with transient ischemic attacks often present with weakness or decreased sensation, and rarely present with LSS. A case–control study involving 313 patients with symptomatic ICA occlusion found that 11% of patients had symptoms of limb shaking.^[11]^ When this occurs, it should be differentiated from focal seizure for adequate treatment. In cases of vascular ischemic changes, limb-shaking symptoms are provoked by postural changes and neck extension, as observed in our patient. A possible mechanism of LSS can be explained by the hypoperfusion theory, which states that transient hemodynamic insufficiency causes brain hypoperfusion.^[11]^ EEG studies of LSS have failed to show epileptiform activity associated with LSS, even though some patients have slow contralateral activity. Most limb-shaking symptoms found in our literature review were transient. However, in our case of total ICA occlusion, continuous, rhythmic left-hand shaking lasted throughout the day and night, was nonsuppressible, and clinically appeared to be an EPC. However, EPC was ruled out because no epileptiform discharge was found on the EEG.

Patients with RA are at high risk for carotid artery stenosis. Therefore, aggressive control of RA and early treatment of CV risk factors could lower morbidity and mortality in these patients. The EULAR recommends that a risk estimate be performed for each patient and has suggested specific DMARDs, such as anti-tumor necrosis factor and methotrexate, for those with high CV risk, especially those with seropositivity, increased inflammatory markers, long disease duration (>10 years), and/or extra-articular manifestations.^[12]^ Glucocorticoid therapy has been suggested as an adjunctive therapy for early RA due to its anti-inflammatory effects. Attempts should be made, however, to maintain short-term use and minimal effective dose due to the side effects associated with chronic use. In our case, the steroid dose was titrated up to 20 mg/day during active RA and poor control and gradually tapered to 5 mg/day after transition to DMARDs and when RA was better controlled. In patients with poorly controlled RA, switching to anti-tumor necrosis factor agents should be considered early in the disease course.

Treatment of severe carotid stenosis or occlusion includes antiplatelet use, revascularization procedures, avoidance of neck massages, maintenance of adequate fluid status (avoiding dehydration), and careful control of blood pressure (avoiding hypotension). Our patient did not undergo aggressive revascularization because of the presence of collateral circulation. Antiepileptic drugs were used because of continuous nonepileptic focal myoclonus. Lacosamide, which acts by slowly inactivating voltage-gated sodium channels, is authorized for focal seizure control and has been effective in EPC. Perampanel, a noncompetitive α-amino-3-hydroxy-5-methyl-4-isoxazolepropionic acid receptor antagonist, has been approved for use in partial-onset seizures mainly as an add-on therapy. In this patient, the combination of these 2 drugs, along with clonazepam, was effective.

We performed a literature search using “Rheumatoid arthritis and stroke” as keywords and a date range from 1994 to 2021. A total of 99 articles were identified, irrelevant articles were removed, and 10 case reports were obtained. Meanwhile, the patients’ sex, age, symptoms, duration and medications for RA, clinical manifestations of stroke, and underlying diseases were recorded from the literature. There were 7 female and 3 male patients, ranging in age from 34 to 80 years. Among the stroke types, there were 9 cases of ischemic stroke and 1 case of intracerebral hemorrhage. The mechanisms of stroke secondary to RA included 2 cases of atlantoaxial joint deformities and 8 cases of chronic inflammation. The results are presented in Table 1.^[5,6,13–20]^

**Table 1:** Clinical manifestations of patients with rheumatoid arthritis and stroke.

Author	Age	Sex	RA medications	RA duration	RA symptoms	Stroke symptoms	Other underlying disease
Kuroki et al^[5]^	78	Female	—	10	Joint deformities	Dysarthria, bilateral, horizontal gaze-paretic nystagmus, dysmmetria	Vertical atlantoaxial subluxation, tuberculosis, and osteoporosis
Garg et al^[6]^	45	Male	—	10	Atlantoaxial dislocation, odontoid erosions	Posterior circulation infarcts	—
Watanabe et al^[13]^	80	Female	—	50	—	Limb weakness and dysarthria	Antiphospholipid syndrome
Cojocaru et al^[14]^	46	Female	Methotrexate, prednisone, and anti-inflammatory drugs	8	Diffuse arthralgias	Left hemiplegia	—
Ohta et al^[15]^	64	Female	prednisolone	7	Fever and arthralgia, episcleritis of the eyes and rheumatoid nodules in the skin	Delirious	—
Cojocaru et al^[16]^	78	Male	—	17	—	Visual disturbances, headache, fever, and gait disturbances	Hypertension, diabetes mellitus, an ischemic stroke 13 y ago, polynodular goiter
Chatzis et al^[17]^	41	Male	—	0	Valvulitis	Left hemiplegia	—
Maeshima et al^[18]^	68	Female	—	15	—	Right hemiplegia	—
Kanazawa et al^[19]^	78	Female	Gold -sol, steroid hormone, and nonsteroidal anti-inflammatory drugs	30	—	Right hemiplegia	Nephrotic syndrome
Nakane et al^[20]^	34	Female	—	—	Vasculitis	Sensory disturbance of left big toe and weakness of right lower limb	—

“—” = not mentioned, RA = rheumatoid arthritis.

## 4 Conclusions

RA is associated with atherosclerotic lesions and stroke, particularly when it is poorly controlled. Continuous hand shaking (nonepileptic focal myoclonus) can be the initial presentation of severe ICA occlusion in patients with poorly controlled RA. Every patient with RA should be surveyed for stroke risk factors and severe ICA stenosis, especially those with poorly controlled RA. If ICA stenosis is detected, carotid stenting and endarterectomy should be considered. Specific antirheumatic agents have been suggested to prevent atherosclerosis and subsequent stroke.

## Author contributions

**Conceptualization:** Li-Min Liou.

**Investigation:** Chun-Yi Tsai, Chiou-Lian Lai.

**Supervision:** Meng-Ni Wu.

**Writing – original draft:** Ching-Fang Chien.

**Writing – review & editing:** Li-Min Liou.
